# Identifying CpG sites with different differential methylation frequencies in colorectal cancer tissues based on individualized differential methylation analysis

**DOI:** 10.18632/oncotarget.17647

**Published:** 2017-05-07

**Authors:** Haidan Yan, Jun He, Qingzhou Guan, Hao Cai, Lin Zhang, Weicheng Zheng, Lishuang Qi, Suyun Zhang, Huaping Liu, Hongdong Li, Wenyuan Zhao, Sheng Yang, Zheng Guo

**Affiliations:** ^1^ Key Laboratory of Ministry of Education for Gastrointestinal Cancer, Department of Bioinformatics, Fujian Medical University, Fuzhou, China; ^2^ Department of Systems Biology, College of Bioinformatics Science and Technology, Harbin Medical University, Harbin, China; ^3^ Department of Medical Oncology, Fujian Medical University Union Hospital, Fuzhou, China; ^4^ Institute of Biomedical Engineering and Instrumentation, Hangzhou Dianzi University, Hangzhou, China

**Keywords:** colorectal cancer, DNA methylation, relative methylation level orderings, differentially methylated CpG sites, biomarkers

## Abstract

A big challenge to clinical diagnosis and therapy of colorectal cancer (CRC) is its extreme heterogeneity, and thus it would be of special importance if we could find common biomarkers besides subtype-specific biomarkers for CRC. Here, with DNA methylation data produced by different laboratories, we firstly revealed that the relative methylation-level orderings (RMOs) of CpG sites within colorectal normal tissues are highly stable but widely disrupted in the CRC tissues. This finding provides the basis for using the *RankComp* algorithm to identify differentially methylated (DM) CpG sites in every individual CRC sample through comparing the RMOs within the individual sample with the stable RMOs predetermined in normal tissues. For 75 CRC samples, *RankComp* detected averagely 4,062 DM CpG sites per sample and reached an average precision of 91.34% in terms that the hypermethylation or hypomethylation states of the DM CpG sites detected for each cancer sample were consistent with the observed differences between this cancer sample and its paired adjacent normal sample. Finally, we applied *RankComp* to identify DM CpG sites for each of the 268 CRC samples from The Cancer Genome Atlas and found 26 and 143 genes whose promoter regions included CpG sites that were hypermethylated and hypomethylated, respectively, in more than 95% of the 268 CRC samples. Individualized pathway analysis identified six pathways that were significantly enriched with DM genes in more than 90% of the CRC tissues. These universal DNA methylation biomarkers could be important diagnostic makers and therapy targets for CRC.

## INTRODUCTION

The frequencies of somatic mutations and copy number aberrations in cancer genomes including colorectal cancer (CRC) genomes are usually very low [1–3], reflecting the molecular heterogeneity of CRC [4, 5]. The extreme molecular heterogeneity of CRC forms a major barrier for therapy, and thus it would be of special importance if we could find common biomarkers besides subtype-specific biomarkers for CRC. Different from somatic mutations and copy number aberrations, DNA methylation aberrations in cancer genomes are widespread in cancer genomes [6, 7], which provides us the possibility to find common epigenetic aberrations in CRC.

Current methods such as Wilcoxon rank-sum test [8] and *T-test* [9] can only identify differentially methylated (DM) CpG sites between a set of cancer samples and a set of normal controls. However, such population-level DM CpG sites cannot tell us the frequencies of CpG sites differentially methylated in patients. Because the DNA methylation levels of CpG sites in a healthy population vary greatly across different individuals, it would be unreasonable to detect DNA methylation states of CpG sites in each cancer sample by comparing with the average methylation level in a set of normal samples [10, 11]. Recently, we found that within-sample relative expression orderings (REOs) of genes are highly stable across different samples of a particular type of normal tissue but widely disrupted in the corresponding cancer samples [12, 13]. Based on this biological phenomenon, we have developed an algorithm, named *RankComp* [12], to identify differentially expressed genes in each cancer tissue by finding those genes whose up- or down-regulations may lead to the disrupted REOs of genes within this cancer sample in comparison with the highly stable REOs of genes predetermined in accumulated normal samples. Because the highly stable REOs of genes predetermined in accumulated normal samples can represent the REOs of genes in every normal tissue, the differentially expressed genes identified by the algorithm for each disease sample are the genes that are differentially expressed in this disease sample compared with its own previous normal state.

In this study, through the analysis of DNA methylation data produced by different laboratories, we showed that the relative methylation-level orderings (RMOs) of CpG sites are also highly stable within normal colorectal samples but widely reversed in CRC tissues. Therefore, we supposed that the *RankComp* algorithm can be used to detect DM CpG sites in cancer samples at the individual-level. Using 75 paired methylation profiles for colorectal cancer tissues and the paired adjacent normal tissues, we firstly evaluated the performance of *RankComp* by evaluating the identified DM CpG sites in each cancer tissue according to the observed DNA methylation level differences (hyper- or hypo-methylation) between the cancer tissue and its adjacent normal tissue. Since the performance of *RankComp* was evaluated based on each paired samples, 75 independent tests were performed. Finally, we detected DM genes and significant deregulated pathways in each of 268 CRC samples from The Cancer Genome Atlas (TCGA) and revealed that there are common DNA methylation biomarkers of CRC, which could be important diagnosis makers and therapy targets for CRC.

## RESULTS

### Performance of *RankComp* for individualized differential methylation analysis

From two independent datasets, GSE42752 and GSE48684, 7320 and 9962 DM CpG sites were detected between cancer and normal groups (*T-test*, FDR < 0.01), respectively. The two lists of DM CpG sites have 6060 overlapped CpG sites, among which 98.69% of the overlaps have the concordant hypermethylation or hypomethylation states in the two datasets. These reproducible DM CpG sites were defined as the population-level DM CpG sites for CRC (Supplementary Table 1). Then, we did individualized analysis of the CpG sites for these population-level DM CpG sites using the *RankComp* algorithm (see Methods).

Firstly, we identified 152,666,734 and 218,691,193 stable CpG site pairs with stable RMOs in at least 99% of the 142 and 82 samples of normal colorectal tissues assayed by the Illumina Human Methylation 27 Beadchip (27K) and 450 Beadchip (450K) arrays (Table 1), respectively. Notably, 90.62% of the stable CpG site pairs in the shorter list were included in the longer list and 99.94% of the overlapped CpG site pairs had the same RMOs patterns for the normal colorectal tissues (binomial test, *p* < 2.2 × 10^–16^). This result suggests that the within-sample RMOs of CpG sites in colorectal normal tissues are highly stable and can be reproducibly detected across different datasets measured by different platforms.

**Table 1:** The DNA methylation profiles analyzed in this study

Dataset	Normal	Tumor	Platform
GSE27130	118	/	27 K
GSE29490	24	/	27 K
GSE42752	41	22	450 K
GSE48684	41	106	450 K
TCGA*	75	75	450 K + 27 K

Note: *represents the paired cancer-normal samples used to evaluate the performance of *Rankcomp*.

**Figure 1:**
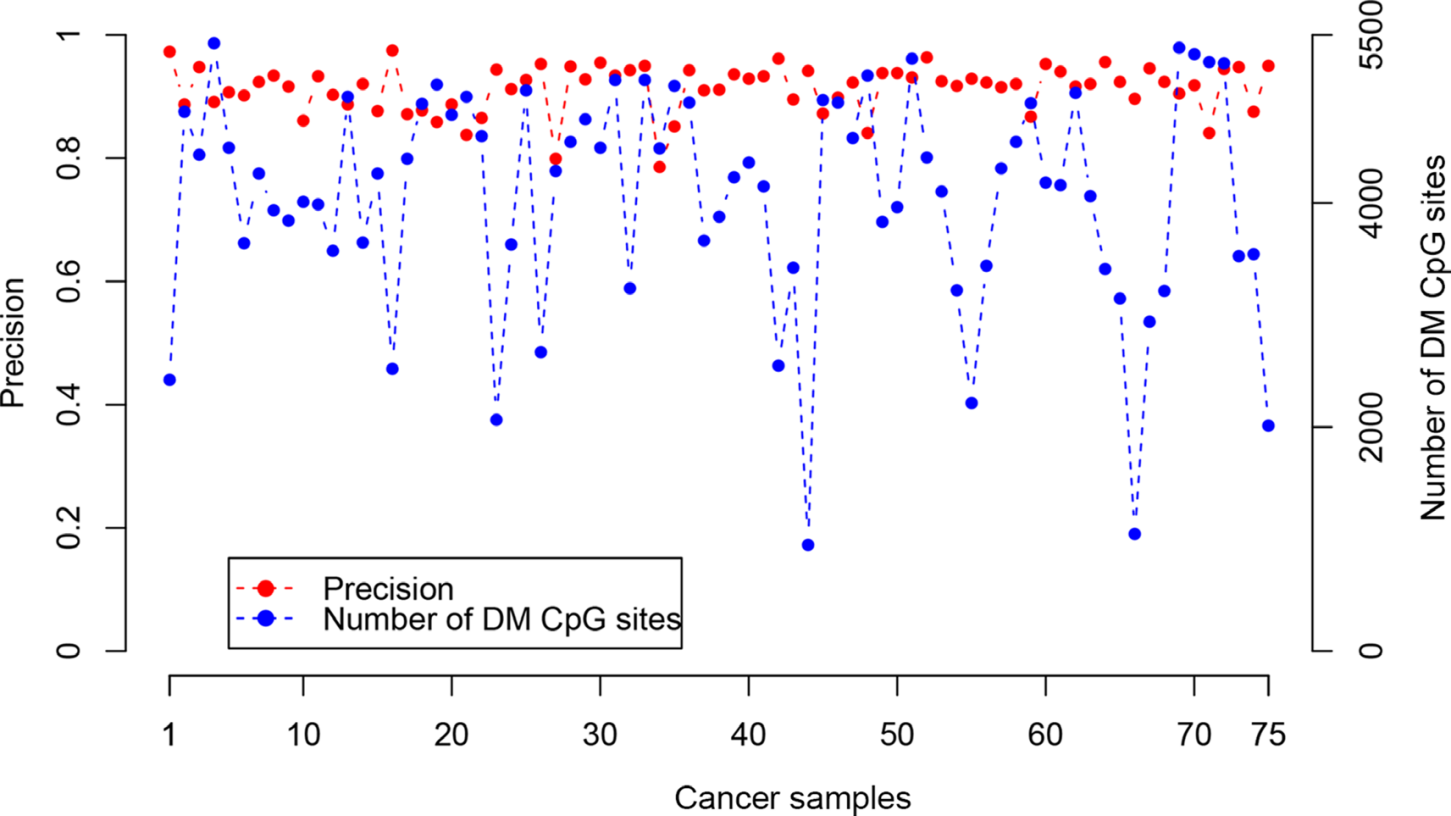
The precision and the number of DM CpG sites detected by *RankComp* for each of the 75 CRC samples with paired adjacent normal tissues from TCGA

Based on the above finding, we evaluated the performance of *RankComp* for detecting DM CpG sites in cancer samples at individual-level, using 75 CRC samples with paired adjacent normal tissues from TCGA. Based on the stable CpG site pairs predetermined in the above 224 normal colorectal tissue samples, averagely 4,062 DM CpG sites per sample were identified with FDR < 0.01. Evaluated according to the observed DNA methylation level differences between each cancer tissue and its adjacent normal tissue, *RankComp* reached an average precision of 91.34% for DM CpG sites detected in individual CRC samples (Figure 1). It suggests that *RankComp* can accurately find DM CpG sites in an individual CRC sample compared with its own previous normal state approximately represented by its paired adjacent normal tissue.

### Universal and subtype-specific DM CpG sites in CRC tissues

Then, we used *RankComp* to identify DM CpG sites for each of the 268 CRC samples from TCGA. A gene was defined as a DM gene in a disease sample if at least one CpG site within its promoter region was identified as a DM CpG site. Genes with inconsistent methylation aberration states (hypermethylation or hypomethylation) within their promoter regions were excluded from the following analyses.

We found 143 genes that were hypomethylated in more than 95% of the 268 CRC samples (Supplementary Table 2). These genes universally altered in CRC might play important roles in CRC genesis and development. For example, *POU5F1* (also known as OCT4), functioning in stem cell pluripotency and embryonic development, was hypomethylated in 98.13% of the 268 cancer samples, which is concordant with the findings that this gene is overexpressed in CRC [14] and may contribute to CRC development [15]. For another example, DNAJB8 was hypomethylated in 97.76% of the 268 CRC samples, which is concordant with the findings that this gene's overexpression can enhance tumorigenicity of CRC cells [16]. Similarly, we found 26 genes that were hypermethylated in more than 95% of the 268 CRC samples (Supplementary Table 2). Because it is well known that hypermethylation of CpG sites in gene promoter regions tend to silence gene expression [17], we further analyzed whether the 26 genes universally hypermethylated in the CRC tissues are frequently deregulated in CRC tissues. Using 16 paired samples of cancer and its adjacent normal tissues with both DNA methylation profiles and gene expression profiles available from TCGA, we found that 14 of the 26 hypermethylated genes had lower gene expression levels in the cancer tissues than in the corresponding adjacent normal tissues in at least 15 of the 16 paired samples (Table 2). Among these 14 genes, five genes (*FLI1*, *IRF4*, *NTRK3*, *SLC6A15*, *KCNQ5*) were known cancer driver genes documented in the F-Census database [18]. For example, *IRF4*, hypermethylated in 99.63% of the 268 CRC samples and downregulated in all the 16 paired cancer tissues, is an important transcript factor for the regulation of interferon-inducible genes and its promoter hypermethylation is a potential biomarker for the diagnosis and therapy of CRC [19]. NTRK3 (also known as TRKC), hypermethylated in 97.76% of the 268 CRC samples, was found to be hypermethylated and downregulated in 15 of the 16 cancer tissues compared with their paired adjacent normal tissues respectively. This gene is a colorectal cancer tumor suppressor gene [20, 21]. Besides the five cancer driver genes, we found that *TMEFF2* (also known as HPP1), hypermethylated in 95.52% of the 268 CRC samples, was hypermethylated and downregulated in all the 16 cancer tissues compared with their paired adjacent normal tissues. It has been reported that hypermethylation of this gene may promote the growth and invasive potential of CRC cancer cells [22, 23]. ADHFE1, hypermethylated in 98.13% of the 268 CRC samples, was hypermethylated and downregulated in all the 16 cancer tissues compared with their paired adjacent normal tissues. The downregulation of this gene may induce the proliferation of CRC cells [24]. These results suggested that these frequently hypermethylated and down-regulated genes could be essential medical targets for CRC.

**Table 2:** Down-deregulation numbers of the 14 frequently hypermethylated genes in 16 CRC samples compared with their paired adjacent normal tissues from TCGA

Gene Symble	Hypermethylation frequency	Down-regulated samples	Gene Symble	Hypermethylation frequency	Down-regulated samples
ADHFE1	98.13%	16	CNRIP1	97.76%	15
TMEFF2	95.52%	16	ZNF134	96.27%	15
IRF4	99.63%	16	PHOX2A	97.01%	15
ZNF132	97.01%	16	FLI1	97.01%	15
NSG1	96.27%	16	NTRK3	97.76%	15
NELL1	97.01%	16	KCNQ5	98.51%	15
SLC6A15	95.15%	15	GPM6A	97.76%	15

**Figure 2:**
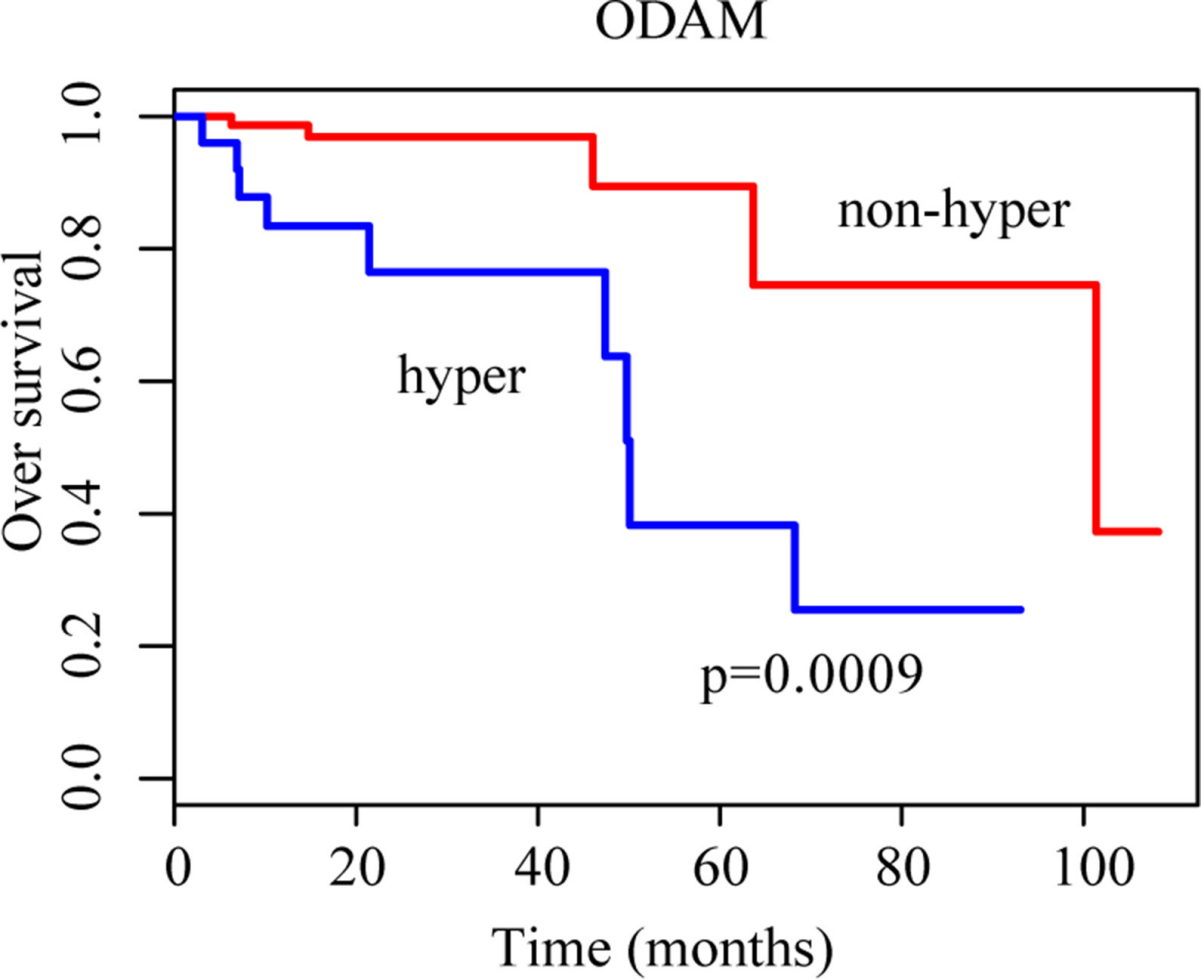
Kaplan-Meier curves for patients grouped based on ODAM methylation The blue and red lines represent patients with and without hypermethylation, respectively.

On the other hand, we found 1288 and 1787 genes that were hypermethylated or hypomethylated in 20–80% of the 268 CRC samples, reflecting the heterogeneity of DNA methylation aberrations across different CRC genomes. Such genes might be subtype-specific genes associated with patients’ prognoses. One important clinical problem for CRC is to develop a signature for predicting prognoses of early-stage (stage I and II) patients undergoing curative surgery since nearly 4% of stage I [25] and 25–30% of stage II [26, 27] patients with curative surgery only will experience relapse. Here, using the overall survival time data of 103 TCGA samples of stage I and II CRC patients with complete surgical resection only, we evaluated whether the patients with and without the deregulation of a gene were significantly different in overall survival (OS) time. Using the univariate Cox proportional-hazards regression model [28], 12 hypermethylated genes and 8 hypomethylated genes were identified to be significantly associated with OS time of stage I and II CRC patients treated with complete surgical resection only (*p-value* < 0.01) (Supplementary Table 3). For example, ODAM was hypermethyled in 24.27% of the 103 stage I-II CRC patients and these patients had significantly shorter OS time (log-rank test, *p* = 0.0009) than the other patients without ODAM hypermethylation (Figure 2). This was consistent with a previous report that down-regulation of ODAM is correlated with decreased overall survival in colorectal cancer since ODAM plays a protective role by inhibiting cells proliferation and metastasis in CRC [29].

### Individualized analysis of pathways for CRC

After identifying DM genes for a disease sample, we were able to detect pathways significantly enriched with the DM genes for this cancer sample.

**Figure 3:**
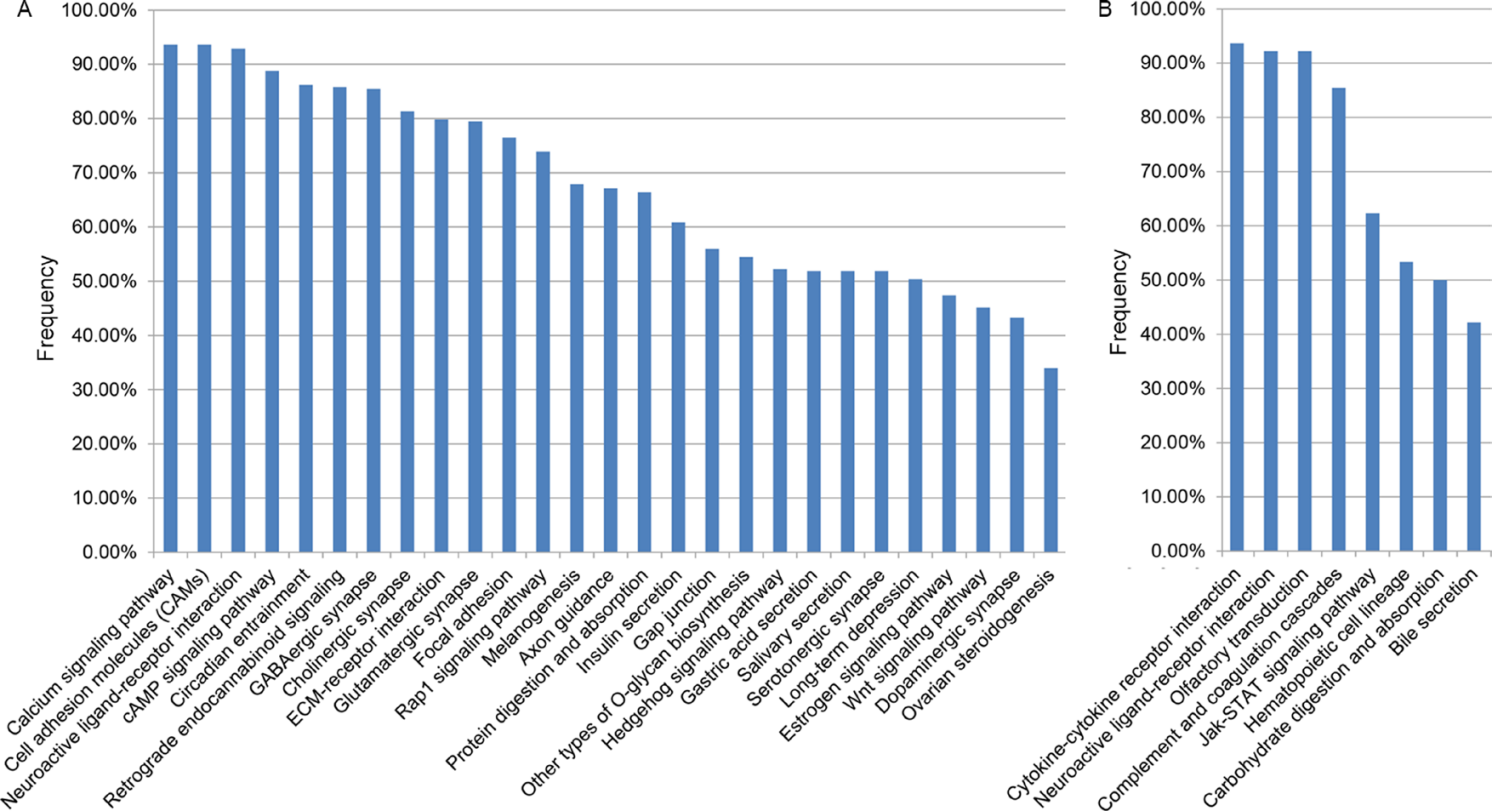
The KEGG pathways separately enriched with hypermethylation (**A**) and hypomethylation (**B**) genes in at least 30% of the 268 TCGA CRC samples.

With FDR < 0.1, 27 pathways were significantly enriched with hypermethylated genes in at least 30% of the 268 CRC samples from TCGA. Especially, three cancer-associated pathways for calcium signaling [30], cell adhesion molecules (CAMs) [31, 32] and neuroactive ligand-receptor interaction [33, 34] were significantly enriched with hypermethylated genes in more than 90% (93.66%, 93.66% and 92.91%, respectively) of the 268 CRC samples (Figure 3A). Similarly, among the eight pathways that were significantly enriched with hypomethylated genes in at least 30% of the 268 CRC samples (Figure 3B), we found that three cancer-associated pathways for cytokine-cytokine receptor interaction (93.66%) [35], neuroactive ligand-receptor interaction (92.16%) [33, 34] and olfactory transduction (92.16%) [36, 37] were significant in more than 90% of the 268 CRC samples. These pathways commonly altered in CRC might be important for studying the mechanisms of CRC.

## DISCUSSION

In this work, we have confirmed that the within-sample RMOs of CpG sites keep highly stable in normal colorectal tissues, which are widely disrupted in CRC tissues. The intrinsic biological phenomena provide a basis for identifying DM CpG sites in CRC samples at individual-level through analyzing the widely disrupted RMOs of CpG sites within every individual cancer sample, taking the predetermined stable RMOs landscape in normal samples as the background. In fact, our result revealed that *RankComp* can accurately identify DM CpG sites for cancer samples at individual-level.

The individualized analysis of DM genes makes it possible to estimate the frequency of an epigenetic aberration in a particular type of cancer such like CRC. The application of the individual-level analysis to 268 CRC samples revealed universal and subtype-specific DM genes and pathways for CRC. Especially, we found 26 and 143 genes that were hypermethylated and hypomethylated, respectively, in more than 95% of the 268 CRC samples. These universally aberrant DNA methylation genes and pathways may be important diagnostic makers and therapy targets for CRC, which deserves our future investigation. On the other hand, genes and pathways with DNA methylation aberrations in a part of the CRC samples could be subtype-specific, which provide hints for dissecting the inter-individual heterogeneity of CRC. Notably, researchers have recently proposed new methods such as CellMethy [38] and CpG_MPs [39] to identify another type of disease methylation biomarkers namely differential concordant methylation of adjacent CpGs, which may provide insight into methylation mechanisms. In line with this direction, we could use a slide window to find adjacent CpGs with concordant methylation aberration status in disease samples after identifying DM CpG sites for individual cancer samples by *RankComp*, and this may deserve our future study.

In summary, the individual-level analysis of DM CpG sites reveals that there are common DNA methylation biomarkers, besides subtype-specific biomarkers, for CRC, which could be important diagnosis makers and therapy targets for CRC.

## MATERIALS AND METHODS

### Data and preprocessing

DNA methylation profiles for colorectal tissues were collected from the Gene Expression Omnibus (GEO) [40] database and The Cancer Genome Atlas data portal (https://tcga-data.nci.nih.gov/docs/publications/tcga/?). As described in Table 1, DNA methylation profiles for 75 paired samples of cancer and adjacent normal tissues from TCGA were used to evaluate the performance of *RankComp*, and the other DNA methylation profiles in normal tissues were used to evaluate the RMOs of CpG sites in normal colorectal tissues. The DNA methylation profiles of 268 samples of CRC were downloaded from TCGA for finding universal and subtype-specific differentially methylated CpG sites in CRC based on individualized differential methylation analysis.

Here, we only analyzed the 25,978 CpG sites measured by both the 27 K array and 450 K array. Using methylated signal intensity (M) and unmethylated signal intensity (U), the DNA methylation level of each probe was calculated by M/(U + M + 100) [41]. The probes were annotated to genes according to the annotation table of 27 K platform.

### Identification of the stable RMOs of CpG sites in normal colorectal tissues

The RMO of two CpG sites (A and B) was defined as stable when their RMO (A > B or A < B in methylation level) was identical in at least 99% of the colorectal normal samples collected from multiple data sources, allowing 1% detection error rate.

To evaluate the reproducibility of stable RMOs of CpG sites between different platforms, we identified two lists of stable CpG site pairs in the normal samples assayed by 27 K and in the normal samples assayed by 450 K, respectively, and then calculated their concordance. If the two lists of stable CpG site pairs shared *k* stable CpG site pairs, among which *s* pairs had the same RMO patterns in the two lists, then the concordance score was calculated as *s/k*. The probability of observing this concordance score by chance was calculated according to the cumulative binomial distribution model [42].

$$P=\;1\;-\;\sum_{I\;=\;0}^{S\;-\;1}\left(\begin{array}{c} k\\ i \end{array}\right){\left(p_{e}\right)}^{i}\;{\left(1\;-\;p_{e}\right)}^{k\;-\;i}$$ (1)

Where *Pe* (*Pe* =0.5) is the probability of the RMO of one CpG site pair shared by the two lists by chance.

### Identification of individual-level DM CpG sites by the *RankComp* algorithm

**Figure 4:**
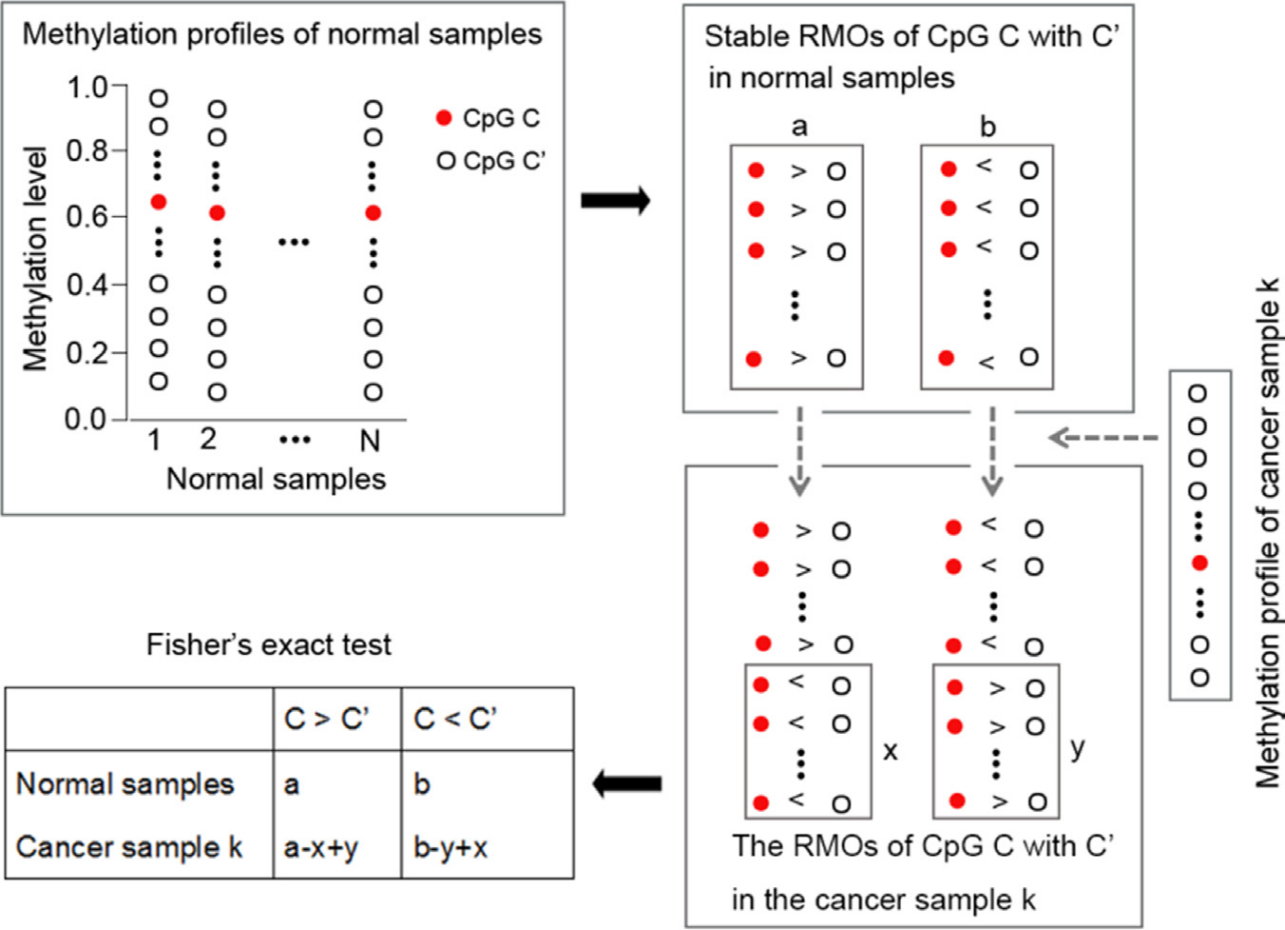
The flowchart of the individual-level DM CpG sites analysis with *RankComp* algorithm

The flowchart of using *RankComp* for detecting individual-level DM CpG sites is shown in Figure 4.

As shown in Figure 4, highly stable CpG site pairs with consistent RMOs in at least 99% of normal samples accumulated from different laboratories were identified, which were next used as normal background. Second, we identified reversal CpG site pairs of each CpG site for a given cancer sample in comparison with their stable RMOs in normal samples. Finally, the Fisher's exact test was used to determine whether a CpG site was differentially methylated in a given cancer sample by testing the null hypothesis that the proportion of reversal CpG site pairs supporting the hypermethylation of this CpG site was equal to the proportion of reversal CpG site pairs supporting the hypomethylation of this CpG site. For a given CpG site *C*, if its methylation level was stably lower (or higher) than that of a CpG site *C’* in the normal samples but this ordering was reversed in a cancer sample, then this reversal CpG site pair could support hypermethylation (or hypomethylation) of *C* in this cancer sample. The detail of the *RankComp* algorithm, previously developed for detecting individual-level differential expression genes, is described in [12]. The software of the *RankComp* algorithm is available at https://github.com/pathint/reoa.

### Performance evaluation of *RankComp*

To ensure the individual-level DM CpG sites to be associated with cancer, we focused on individualizing the CpG sites that were found to be differentially methylated at the population-level. Thus, the *T-test* was used to detect population-level DM CpG sites between cancer samples and normal controls using two independent datasets, respectively, and the DM CpG sites consistently detected from the two independent datasets were defined as the population-level DM CpG sites for CRC. The *p*-values were adjusted using the Benjamini-Hochberg procedure [43].

The DNA methylation profiles for 75 CRC tissue samples with paired adjacent normal tissues were used to evaluate the performance of *RankComp*. We firstly identified DM CpG sites in each of the 75 cancer samples by *RankComp* using DNA methylation data on cancer samples alone. We then evaluated the precision of individual-level DM CpG sites for each of the cancer samples using the observed methylation level differences (hypermethylation or hypomethylation) between this cancer sample and its paired adjacent normal sample as the golden standard. The underlying assumption of this evaluation is that the previously normal state of a cancer tissue could be approximately represented by the adjacent normal tissue of the cancer tissue. For a cancer sample, if the hypermethylation or hypomethylation states of DM CpG sites detected by *RankComp* are consistent with the golden standard, then they are defined as true positives (TP); otherwise, false positives (FP). The precision of the DM CpG sites detected for each CRC sample is calculated as the positive predictive value: TP/(TP + FP). Since the performance of *RankComp* was evaluated based on each paired samples, 75 independent tests were performed.

### KEGG pathways

For pathway enrichment analysis, data of 234 pathways covering 5981 unique genes was downloaded from the Kyoto Encyclopedia of Genes and Genomes (KEGG) (Release 58.0) [44]. The hypergeometric distribution model was used to determine the biological pathways that were significantly enriched with hyper- and hypomethylated genes, respectively [45]. The *p*-values were adjusted using the Benjamini-Hochberg procedure [43].

## SUPPLEMENTARY MATERIALS AND TABLES





## Abbreviations

CRC: colorectal cancer
RMOs: relative methylation-level orderings
DM: differentially methylated
TCGA: The Cancer Genome Atlas
27 K: Illumina Human Methylation 27 Beadchip
450 K: Illumina Human Methylation 450 Beadchip
TP: true positives
FP: false positives
