# Supplementary material for: Identifying CpG sites with different differential methylation frequencies in colorectal cancer tissues based on individualized differential methylation analysis
Source: Oncotarget. 2017 May 7;8(29):47356–64. doi: 10.18632/oncotarget.17647 (PMC5564570; doi:10.18632/oncotarget.17647)
Supplement: Supplementary file 1 [file oncotarget-08-47356-s001.pdf]

Identifying CpG sites with different differential methylation frequencies in colorectal cancer tissues based on individualized differential methylation analysis

Supplementary Materials

Supplementary Table 1: Population-level DM CpG sites identified in two independent datasets for colorectal cancers

Tissue	Dataset	Num_hyper	Num_hypo	Con_ hyper	Con_hypo	Con_score
Colorectum	GSE42752	3484	3836	2754	3227	98.69%
	GSE48684	4356	5606			

Note: Num_hyper and Num_hypo represent the numbers of hypermethylated and hypomethylated CpG sites, respectively. Con_hyper and Con_hypo represent the numbers of concordant hypermethylated and hypomethylated CpG sites. Con_score is the concordance score.

Supplementary Table 2: 143 genes that were hypomethylated in more than 95% of the 268 CRC samples. See Supplementary_Table_2

Supplementry Table 3: 12 hypermethylated genes associated with OS of early stage CRC patients treated with surgical resection only

Gene ID	Gene symbol	Average OS times(month)of patients with hypermethylated genes	Proportion of patients with hypermethylated genes	Average OS times(month) of patients without hypermethylated genes	*P*-value
54959	ODAM	28.1480	0.2427	28.9769	0.0009
5100	PCDH8	30.9440	0.5146	26.4773	0.0030
2587	GALR1	28.2230	0.7184	30.1862	0.0043
6247	RS1	23.6515	0.2136	30.1675	0.0044
254427	PROSER2	29.4410	0.3786	28.3703	0.0048
25861	WHRN	30.3016	0.7864	23.1576	0.0056
79034	C7orf26	32.4606	0.6990	20.2172	0.0072
79575	ABHD8	31.5061	0.4757	26.2981	0.0074
160728	SLC5A8	27.9383	0.3883	29.3074	0.0092
260436	FDCSP	32.1017	0.3883	26.6640	0.0096
1816	DRD5	30.0129	0.4757	27.6531	0.0097
664	BNIP3	20.3984	0.2039	30.9211	0.0100

Eight hypomethylated genes associated with OS of early stage CRC patients treated with surgical resection only

Gene ID	Gene symbol	Average OS times(month) of patients with hypomethylated genes	Proportion of patients with hypomethylated genes	Average OS times(month) of patients without hypomethylated genes	*P*-value
942	CD86	28.7430	0.7379	28.8679	0.0033
7201	TRHR	28.6516	0.7961	29.2603	0.0043
115352	FCRL3	27.8721	0.8350	33.3471	0.0045
922	CD5L	29.0502	0.8252	27.4796	0.0052
2554	GABRA1	35.0136	0.2621	26.5596	0.0057
90655	TGIF2LY	30.2346	0.7573	24.2240	0.0063
26160	IFT172	29.3417	0.3495	28.4716	0.0077
51438	MAGEC2	28.3498	0.7864	30.3439	0.0081
